# Pharmacomicrobiomics: Exploiting the Drug-Microbiota Interactions in Antihypertensive Treatment

**DOI:** 10.3389/fmed.2021.742394

**Published:** 2022-01-19

**Authors:** Hui-Qing Chen, Jin-Yu Gong, Kai Xing, Mou-Ze Liu, Huan Ren, Jian-Quan Luo

**Affiliations:** ^1^Department of Pharmacy, The Second Xiangya Hospital, Central South University, Changsha, China; ^2^Institute of Clinical Pharmacy, Central South University, Changsha, China; ^3^Department of Clinical Pharmacology, Xiangya Hospital, Central South University, Changsha, China

**Keywords:** pharmacomicrobiomics, antihypertensive drugs, gut microbiota, precision medicine, interaction

## Abstract

Hypertension is a leading risk factor for cardiovascular diseases and can reduce life expectancy. Owing to the widespread use of antihypertensive drugs, patients with hypertension have improved blood pressure control over the past few decades. However, for a considerable part of the population, these drugs still cannot significantly improve their symptoms. In order to explore the reasons behind, pharmacomicrobiomics provide unique insights into the drug treatment of hypertension by investigating the effect of bidirectional interaction between gut microbiota and antihypertensive drugs. This review discusses the relationship between antihypertensive drugs and the gut microbiome, including changes in drug pharmacokinetics and gut microbiota composition. In addition, we highlight how our current knowledge of antihypertensive drug-microbiota interactions to develop gut microbiota-based personalized ways for disease management, including antihypertensive response biomarker, microbial-targeted therapies, probiotics therapy. Ultimately, a better understanding of the impact of pharmacomicrobiomics in the treatment of hypertension will provide important information for guiding rational clinical use and individualized use.

## Introduction

Hypertension is recognized as a leading risk factor for cardiovascular disease worldwide (1). It has become a healthcare burden and the main cause of mortality from stroke, heart failure and myocardial infarction (2). Approximately 1.5 billion people (one-third of the world's adult population) had hypertension, traditionally defined as a clinic blood pressure (BP) of ≥140/90 mmHg (3, 4). During the past 30 years, the treatment of hypertension has advanced considerably, such as lifestyle intervention and antihypertensive drug therapy (5, 6). Patients with hypertension usually have to take antihypertensive drugs continuously, as a cure is not available (7, 8). There are 23 kinds of antihypertensive agents available with over 100 different types of antihypertensive drugs, such as β-blockers (BB), diuretics, calcium channel blockers (CCB), angiotensin II receptor blockers (ARB) and angiotensin-converting enzyme inhibitors (ACE-I), Ram (9). However, according to the recent guidelines for hypertension management, nearly 40% of patients still did not achieve their BP target (10–12), meaning some patients cannot benefit from medication. Therefore, insights into the potential factors that affect the efficacy of antihypertensive drugs *in vivo* are urgently needed to maximize clinical response.

In recent years, the gut microbiota has been regarded as an “invisible organ” and affected human health and disease development (13–15). Although pharmacogenomics and pharmacogenetics have been at the forefront of research examining the influence of individual genetic background on drug response, the focus of research has also been extended to the potential mechanism of gut microbiota to drug efficacy (16). The human gut microbiota has a huge gene bank that encodes approximately 100 times more genes than the human genome. Thus, gut microbial communities may become an indispensable part of the personalized medical movement in the future (17, 18). The study of the bidirectional effects between drugs and microbiota, termed pharmacomicrobiomics, is an extension of pharmacogenomics (19, 20). Recent developments in genome sequencing technologies and bioinformatics can not only dissect gut microbiome composition and their genes in detail, but also investigate the influence of variations within the human gut microbiota on antihypertensive drugs (21–23). These studies have promoted the progress of personalized medicine in the hypertension treatment.

In this review, we clarify the recent research in a new field of pharmacomicrobiomics by the revealing reciprocal interplay between the gut microbiota and the drug. We also describe the dynamic interaction between the gut microbiome and specific antihypertensive drugs and analyze the effect of alteration in the composition and function of gut microbiome on antihypertensive drug action. Finally, we discuss the strategy to incorporate pharmacomicrobiomics into the frontier of precision medicine in hypertension.

## The Interaction of Drug and Gut Microbiota

**Figure 1:**
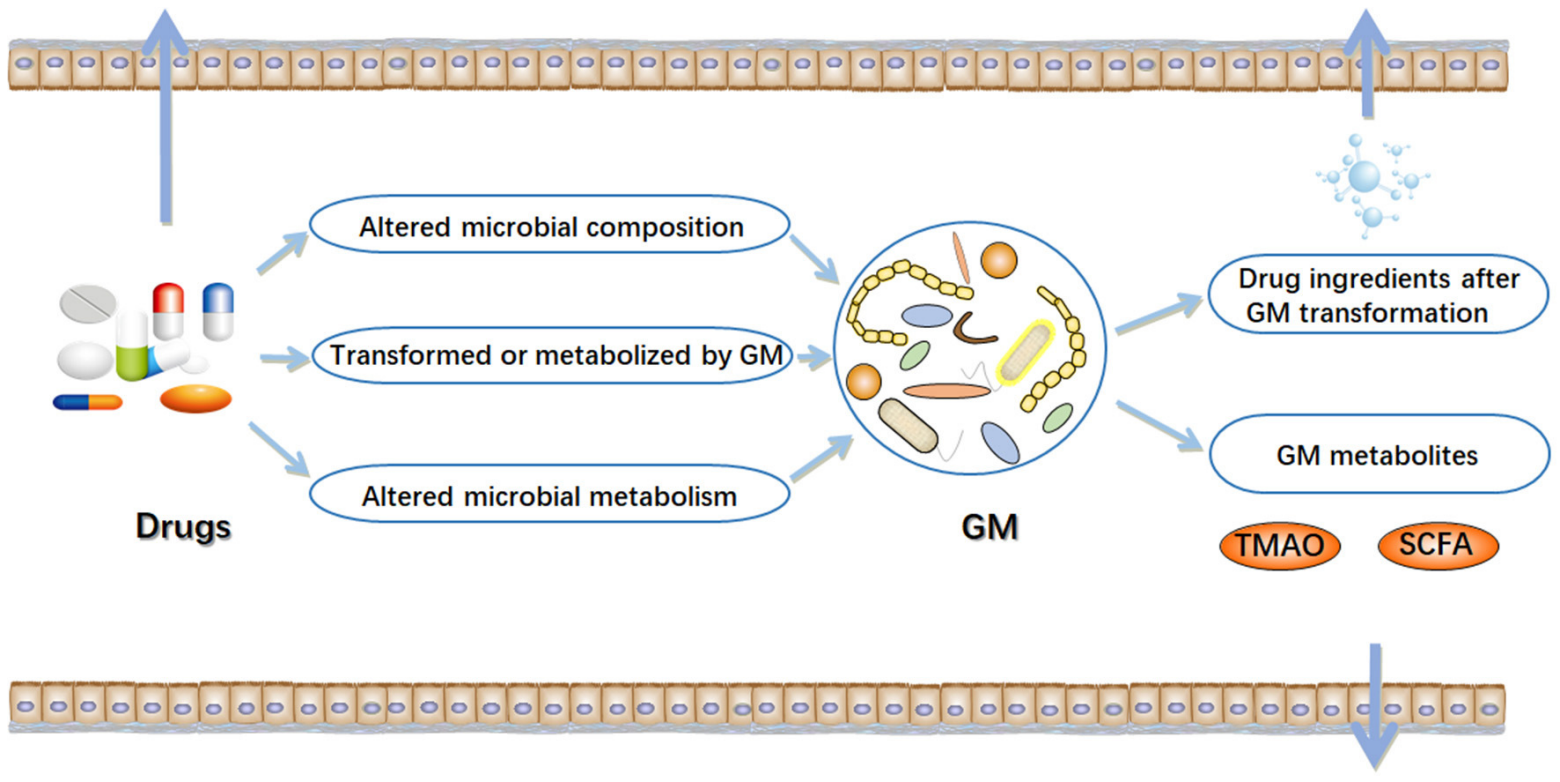
Interactions between drugs and gut microbiome (GM). In the intestinal tract, there are complex interactions between drugs and microorganisms. On the one hand, drugs can result in alterations in the composition and function of gut microbiome. On the other hand, gut microbiome may alter chemical structure of drugs, and directly or indirectly affect drug efficacy. TMAO, trimethylamine N-oxide; SCFA, short-chain fatty acids. Arrow mark indicates Drugs, GM metabolites and drug ingredients after GM transformation are transferred outside the gut.

In the early stages of life, humans begin to ingest various xenobiotics, such as a multitude of drugs and dietary molecules (24). After birth, humans are rapidly settled by trillions of microorganisms, most of which eventually live in their gastrointestinal tract (25, 26). The gastrointestinal tract has a micro-environment suitable for the growth of microorganisms, making it an excellent habitat for the metabolic potential of microbial community (27). The microbiota can provide nutrients to the host, assist in metabolism, and directly affect the development of the immune system and its ability against pathogens (28–30). In particular, variability in the component and metabolic capacity of the gut microbiome has a major role in determining toxicity and clinical efficacy of drugs. Enzyme activity in gut microbial communities can alter the chemical structure of drugs, thereby affecting their bioavailability (31–33). Drugs also alter the composition and function of gut microbial community, thereby changing intestinal microenvironment and affect microbial metabolism (16) (Figure 1). The interaction between drugs and the gut microbiome has garnered growing interest in the field of precision medicine.

### Gut Microbiome Influences Drug Response

Oral drug administration is the most complicated path of delivery. When oral medications move through the upper gastrointestinal tract and small intestine and enter the large intestine, they contact thousands of microbial species (16). The gut microbiota can synthesize a series of enzymes involved in drug metabolism, including oxidase, hydrogenase, etc. These enzymes can activate, inactivate or reactivate drugs by structural change of their components (34–36). Due to the high metabolic activity of gut microbiota, it is considered a metabolic organ with the same metabolic capacity as the liver (37). The enzymes in the liver predominantly perform oxidative and conjugative reactions to generate metabolites with a higher polarity or molecular mass, while the gut microbes usually conduct hydrolytic and reductive reactions to produce metabolites with a lower polarity and relative molecular mass (32, 38). In addition, chemical reactions involved in gut microbes include dehydrogenation, debenzene, decarbonization, deethylation, desulfurization and acetylation (39, 40). So far, over 30 drugs have been identified as gut bacteria substrates (41). The direct influence of microorganisms on drug response is that the gut microbiota affects the bioavailability of drugs by transforming their chemical structures (39).

### The Effect of Drugs on Gut Microbiome

When considering the influence of gut microbiome on drug response, the pharmacological effect of drugs on gut microbiome composition should also be considered (35). Recently, it was demonstrated that about 24% of 1,000 tested drugs inhibited at least one bacterial strain from gut microbiome *in vitro* (42–44). Drugs may affect host metabolism or clinical efficacy by regulating gut microbiome composition or its function (45–47). For instance, antibiotic-induced microbiota dysbiosis not only increases susceptibility to infection but also impairs immune homeostasis. It is also the leading cause of Clostridium difficile infection, and the excessive reproduction of this bacteria causes severe intestinal inflammation (48, 49). Beyond antibiotics, population-based studies have revealed that other commonly used drugs can also affect the gut microbiome (50–52). Metformin, an anti-diabetic drug, increases the abundance of Akkermansia muciniphila in the intestine, promotes short-chain fatty acid bacteria in the body, and plays a positive therapeutic effect on improving insulin resistance and glucose homeostasis (45, 53).

## Gut Microbiome and Antihypertensive Drugs

Researchers have recently discovered a close relationship between gut microbiome and antihypertensive drugs. The first-line antihypertensive drugs include CCB, ARB, ACE-I, and BB etc. Different species of antihypertensive drugs have significant differences in their mechanisms for lowering blood pressure, and their interactions with gut microbiome are also different (22). Next, we introduce the interaction between gut microbiome and several specific antihypertensive drugs (Table 1).

**Table 1:** Drug-microbiome interactions of antihypertensive drugs.

**Drug**	**Relationship**	**Animal/human**	**Effect on PK**	**Effect on GM**	**Refs**
Amlodipine	GM affects drug	SD rats	↓ AUC ↓ M1 ↓ T_max_ ↓ C_max_	N/A	(54)
Diltiazem	GM affects drug	Sterile mice	↓ F	N/A	(55)
Losartan	Drug affects GM	SHR, WKY	N/A	↑ Akkermansia ↑ Pedobacter ↑ Verrucomicrobiacea ↓ Lactobacillus ↓ Lactobacillaceae	(56)
Captopril	Drug affects GM	SHR, WKY	N/A	↑ Tenericutes ↑ Actinobacteria ↑ Proteobacteria ↑ Firmicutes ↓ Bacteroidesare	(57)
Captopril	Drug affects GM	SHR, WKY	N/A	↑ Dehalobacterium ↑ Oscillospira ↑ Roseburia ↑ Coprococcus	(58)
Benazepril	Drug affects GM	SHR, WKY	N/A	↓ Aggregatibacter ↓ Lactobacillus ↓ Veillonella ↑ Prevobacterium	(59)
Enalapril	Drug affects GM	Wistar rats	N/A	↓ Collinsella ↑ Clostridium	(59)
Metoprolol	Drug affects GM	Hypertensive patients	N/A	Metabolites (GM): ↑ Methyluric acid ↑ Hydroxyhippuric acid ↑ Hippuric acid	(60)
Nifedipine	GM interacts with drug	Wistar rats	↓ AUC ↓ CL ↓ T_max_	↓ Enterobacteriaceae	(55)

*GM, gut microbiota; PK, pharmacokinetics; SD rats, sprague dawley rats; SHR, spontaneously hypertensive rats; WKY, wistar kyoto rats; N/A, not available; AUC, area under the concentration–time curve; M1, amlodipine metabolite; T_max_, peak time; C_max_, maximum concentration; F, relative bioavailability; CL, plasma clearance; ↓, decreased; ↑, increased*.

### CCB

#### Amlodipine

Amlodipine is a typical CCB, which is one of the most commonly used prescription drugs for hypertension treatment. Amlodipine is relatively well-absorbed from the gastrointestinal tract with a bioavailability of approximately 60% following oral administration (61). Patients with hypertension usually take amlodipine with other drugs, and one of the most likely co-administered drugs is antibiotics.

**Figure 2:**
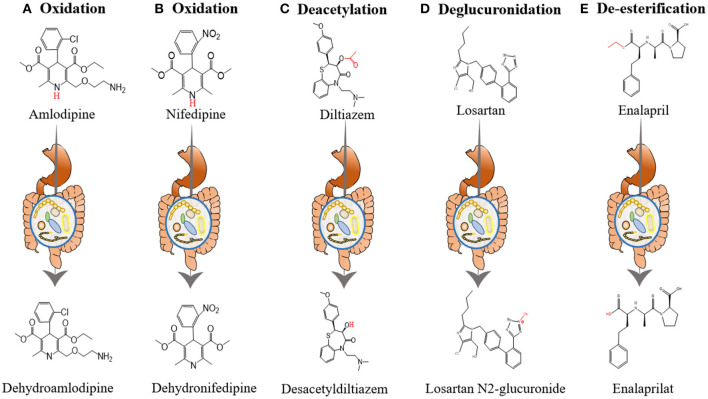
The influence of gut microbiome on the metabolism of antihypertensive drugs. **(A)** Oxidation of amlodipine. Amlodipine is converted into dehydroamlodipine through the oxidation reaction of gut microbiome. **(B)** Oxidation of nifedipine. Biotransformation of nifedipine produces dehydronifedipine through gut microbiome. **(C)** Deacetylation of diltiazem. Diltiazem is converted into desacetyldiltiazem through the action of gut microbiome. **(D)** Deglucuronidation of losartan. Losartan is converted into losartan N2-glucuronide through the action of gut microbiome. **(E)** De-esterification of enalapril. Enalapril is converted into enalaprilat through the de-esterification reaction of gut microbiome.

Yoo et al. investigated the interactions between amlodipine and co-administered antibiotics associated with changed metabolic activities of gut microbiome (54). They incubated amlodipine with human and rat feces. As the incubation time increased, the remaining amlodipine gradually decreased and the formation of metabolites increased. At 24 and 72 h after incubation, the residual amlodipine decreased by 8.9 and 21.3%, respectively. The spectrometric analysis identified structures of the metabolites produced by gut microbiome in amlodipine (Figure 2A), which is a pyridine metabolite (M1), mainly produced by oxidation reaction and formed by liver metabolic enzymes (62). The clearance rate of amlodipine indicated that gut microbiome may participate in amlodipine biotransformation, affecting the drug's pharmacokinetics.

In addition, the researchers measured amlodipine plasma concentrations in control and antibiotic-treated rats. The area under curve (AUC) values of amlodipine in control and ampicillin-administered rats were 922.6 ± 266.7 and 1,311.5 ± 238.4 ng·h/mL, respectively. Therefore, antibiotics may affect the gut microbiome, reducing amlodipine metabolism by the intestinal microbiome and ultimately increasing drug bioavailability.

#### Nifedipine

It is well-recognized that nifedipine, a non-polar drug, can be basically absorbed by the human gastrointestinal tract (63, 64). Nifedipine is likely to be transformed by gut microbiota in this process (28). To the best of our knowledge, there are many external factors that affect the gut microbiome. For instance, the low-oxygen environment of the plateau can affect the composition of the gut microbiome (65).

Zhang et al. investigated the influence of plateau hypoxia-mediated gut microbiome dysregulation on metabolic conditions and therapeutic effect of nifedipine (66). They divided Wistar rats into plateau, plain, and amoxicillin-treated plain groups, collected rat feces for analysis, and observed alteration in the number of representative gut microbiomes microscopically. Compared with plain rats, the number of Enterobacteriaceae was reduced in amoxicillin-treated plain group. In addition, plateau group rats appeared Bacillus in comparison to the amoxicillin-treated plain group. These results demonstrated that hypoxia at high altitudes and amoxicillin treatment can alter the gut microbiome, thereby affecting the metabolism of drugs *in vivo*.

In addition, the researchers evaluated changes in the metabolic activity and oxidation products of nifedipine after incubation with rat feces. After 12 h of incubation, nifedipine levels decreased by 53.72% in the plain group, 34.79% in the plateau group, and 42.57% in the amoxicillin-treated group, and the metabolic nifedipine percentage were 23.14, 10.84 and 16.67%, respectively. Pharmacokinetics results showed that compared with the plain group, the AUC of amoxicillin-treated plain group was significantly increased by 39.10%, while the peak time (T_max_) and plasma clearance (CL) were decreased by 48.91 and 34.71%, respectively. Tandem mass spectrometry (MS/MS) analysis identified the structure of metabolites produced by the gut microbiome in nifedipine, and oxidized nifedipine is the main metabolite (Figure 2B). Consequently, gut microbial activity could change the bioavailability and pharmacokinetics of drugs, and this consideration can be extended to assess the efficacy of drugs.

#### Diltiazem

Diltiazem, a calcium channel blocker, works by relaxing the smooth muscles of the arterial wall and mainly treats angina pectoris and hypertension (67). After oral administration of diltiazem, a potential complicating factor in its pharmacokinetic and pharmacodynamic characteristics is that there are two metabolites in plasma, desacetyldiltiazem, and N-desmethyldiltiazem. Both of these metabolites have important pharmacological activity in animal tissues (68, 69).

Zimmermann et al. revealed that when diltiazem is administered orally, the vasodilator diltiazem can be metabolized to diacetyl diltiazem by human gut microorganism Bacteroides thetaiotaomicron. This strain was used as an exemplary source species because its gene product BT4096 can metabolize diltiazem through deacetylation (Figure 2C). The researchers colonized sterile mice with Bacteroides thetaiotaomicron wild-type or BT4096-deletion strains and administered diltiazem orally. The levels of drug and metabolite in the intestine demonstrate that BT4096 is required for deacetylation of diltiazem and its metabolites. In the human intestine, diltiazem is converted to its deacetylated metabolites by removing the acetyl group from parent drugs through GDSL/SGNH series hydrolase BT4096. By repeating oral administration, bacteria's impact on diltiazem metabolism is further enhanced. This also demonstrates the critical role of a single gut bacterial in drug metabolism (55).

#### ARB (Losartan)

Losartan is recommended as one of the most commonly used antihypertensive drugs. Recent studies indicated that the hypotensive effect of losartan is closely related to gut microbiota (56) (Figure 2D). Robles-Vera et al. used losartan to treat spontaneously hypertensive rats (SHR) and Wistar Kyoto rats (WKY) for 5 weeks. After treated with losartan for 5 weeks, they found that most of the abnormal indexes of SHR improved and were close to WKY. For example, losartan-treated SHR increased the abundance and diversity of gut microbiome and restored the Firmicutes/Bacteroidetes (F/B) ratio. It also increased the percentage of anaerobic bacteria in SHR. In addition, losartan treatment increased mRNA levels of zonula occludens-1 (ZO-1) and occludin in the colon of SHR. And they also found reduced expression of tyrosine hydroxylase (TH) in losartan -treated SHR, a key enzyme involved in noradrenaline production and noradrenaline content in the intestine. These changes improved intestinal integrity and intestinal sympathetic tone in SHR.

Losartan may elicit alterations in gut microbiome through three mechanisms. First, losartan may elicit changes in microbiome by promoting the production of α-defensins. Microbiota components are recognized by Toll-like receptors expressed by intestinal epithelial cells and trigger the production and secretion of defensins. Compared with WKY, mRNA levels of defensins in SHR colon samples were changed, which may also be related to microbiota alterations of this hypertensive rat. Losartan treatment restores the expression of defensin in SHR to a level similar to that of WKY. Second, losartan may cause changes in microbiome by improving the intestinal integrity of SHR. Changes in the compositions of intestinal flora are related to intestine's integrity (70). The epithelial cells of digestive tract of mammals form a tight barrier in the intestine, contributing to the hypoxic environment in the lumen (71). The content of anaerobic bacteria in SHR feces is reduced, which is related to intestinal integrity loss. Losartan-treated SHR showed an increase in colon integrity and a strict anaerobic bacteria ratio comparable to WKY. Finally, losartan may cause changes in microbiome by reducing sympathetic tone activity in the intestine. The intestinal sympathetic drive is a crucial regulator of intestinal integrity and microbiota composition. Increased intestinal sympathetic drive (increased tyrosine hydroxylase expression and norepinephrine accumulation) is related to SHR intestinal integrity loss (72). Losartan treatment reduces sympathetic nerve activity in the colon, improves intestinal integrity and microbial dysbiosis. Overall, losartan treatment reduced gut dysbiosis in SHR and significantly altered the composition and ratio of host gut microbiota.

### ACE-I

#### Captopril

Captopril is the first generation of ACE-I. It can reduce blood pressure by inhibiting renin-angiotensin system (RAS) at both central and peripheral sites (73). Numerous studies indicate that captopril continues to exert antihypertensive effects after discontinuation (74–76). Captopril not only has a long-lasting antihypertensive effect but also possesses a close relationship with gut microbiome composition.

Yang et al. used captopril to treat SHR and WKY for 4 weeks, followed by withdrawal for 16 weeks (57). They used 16S rRNA gene sequencing method to analyze gut microbial composition of these rats. After captopril treatment, the blood pressure of SHR dropped and the amounts of bacterial spores increased, such as Parabacteroides, Mucispirillum and Allobaculum. Compared with WKY, the changes in gut microbiome of SHR in the 4th week included significant enrichment of Tenericutes, Actinobacteria, Proteobacteria, and Firmicutes and a decrease in Bacteroides. This phenomenon resulted in increased trends in the evenness in SHR at the 4th week. Notably, these increasing trends were observed in the 8th week of no CAP treatment. This phenomenon may be due to captopril continuing to affect neuronal activity. Compared with the control WKY, the neuronal activity of the posterior pituitary in SHR increased by 52.7%. After 4 weeks of captopril treatment, this increase was reduced to a level equivalent to WKY and maintained at the 8th week. Therefore, captopril inhibits the neuroinflammation of the autonomic nervous area and weakens the sympathetic driving force of intestinal overactivation (77, 78), thereby balancing the intestinal flora.

In addition, recent research indicates that maternal captopril treatment may have a long-lasting antihypertensive effect by reshaping the gut microbiome and enhancing intestinal pathology and permeability, thereby rebalancing the dysfunctional intestinal-brain axis in male offspring (58). For instance, the class Clostridia and order Clostridiales were richer in SHR of maternal captopril. Compared with pregnant SHR, the order Clostridiales (Dehalobacterium, Oscillospira, Roseburia, and Coprococcus) in gut microbiome of pregnant SHR using captopril showed higher average abundance. Therefore, captopril may affect intestinal bacteria growth in the body and flora composition, thereby changing the drug efficacy (79).

#### Benazepril

Benazepril is the second generation of ACE-I, mainly used to treat cardiovascular diseases such as congestive heart failure and arterial hypertension. Benazepril metabolism is mainly in the liver. It is hydrolyzed into diacid benazeprilat through ethyl ester (80, 81). Recently, a research experiment compared the feces of the SHR group, the WKY group, and the SHR group treated with benazepril (82). The results showed that the composition ratio of gut microbes in SHR after drug treatment changed significantly. From the gate level, Proteobacteria in the gut of SHR is higher than that of WKY, while SHR treated with benazepril reversed this trend. According to the genus level analysis, Streptococcus genus in the gut of SHR is higher than that of WKY, but Benazepril treatment reduces the proportion of this microbiota in the gut microbiota. Furthermore, benazepril treatment also reduced Aggregatibacter, Lactobacillus and Veillonella, and slightly increased the proportion of Prevotella in gut microbiome. According to the analysis of Ace index, Category index, Simpson index and Chao1 index, the abundance and diversity of microbial community in the intestinal tract of SHR tend to WKY after benazepril treatment. Therefore, benazepril can promote the restoration of gut microbiome structure in SHR.

#### Enalapril

Enalapril, the second generation of ACE-I, is an important drug for treating hypertension. Recent experimental studies have manifested that enalapril treatment can reduce blood trimethylamine N-oxide (TMAO) levels (59) (Figure 2E). Plasma TMAO mainly originates from intestinal bacteria metabolism and positively correlates with cardiovascular disease risk (83–85). The intestinal microbes in the body, including Proteus, Desulfovibrio, Lactobacillus, Collinsella and Clostridium, produce some methylamines from lecithin and dietary choline (86, 87). Part of these methylamines is absorbed into the blood and excreted with sweat, urine and other forms. Therefore, the plasma TMAO level depends on many factors, such as diet, microbiota composition and other factors, etc. (88, 89).

Konop et al. studied whether enalapril treatment reduces the blood TMAO level by changing the composition and ratio of gut microbiome (59). They divided Wistar rats into enalapril treatment groups (5.29 ± 0.5 mg/kg in the low-dose group and 12.6 ± 0.4 mg/kg in the high-dose group) and the control group, and analyzed the feces of each group. Compared with controls, Collinsella content in the intestine of enalapril-treated rats was slightly decreased, while Clostridium content was slightly increased. In comparison to controls, Desulfovibrio content was essentially unchanged in the low-dose group but slightly increased in the high-dose group. All research groups showed similar diversity by data analysis, and gut microbiome composition of each group has little difference. However, compared with controls, the plasma TMAO level of rats treated with enalapril was significantly reduced. This indicated that enalapril affects plasma TMAO levels. In addition, 24-h urinary excretion of trimethylamine (TMA) and TMAO increased in enalapril-treated rats. It indicated that enalapril may be involved in controlling methylamines excretion with urine. Simultaneously, enalapril may also reduce the level of TMAO by affecting the metabolic activities of the intestinal flora.

#### Beta-Blockers (Metoprolol)

Metoprolol is the most commonly prescribed BB used to treat cardiovascular diseases, including coronary artery disease, hypertension, and heart failure. Metoprolol is metabolized by a saturable metabolic pathway, namely hepatic cytochrome2D6 (CYP2D6). This drug is mainly metabolized to O-desmethylmetoprolol and α-hydroxymetoprolol (90). Approximately 85% of metoprolol and related metabolites are excreted in urine, making it an ideal object for monitoring (91).

Brocker et al. analyzed the urine of patients taking metoprolol through metabolomics data. They discovered elevated levels of methyluric acid, hydroxyhippuric acid, and hippuric acid in the urine of hypertensive patients after taking metoprolol orally. These three compounds are considered to be metabolites of gut microbiome (60). Methyluric acid is a microbial-dependent metabolite; Hippuric acid is formed by the conjugation of glycine and benzoic acid through gut microbial metabolism and hydroxyhippuric acid is a microbial-derived end product, both of which originate from the polyphenol metabolism of the gut microbiota. These compounds reflect gut microbiota composition (92, 93). It indicates that long-term metoprolol treatment may affect gastrointestinal tract microbial composition and diversity (94). Moreover, metagenomics analysis of stool samples from patients with atherosclerotic cardiovascular disease revealed that metoprolol treatment was positively correlated with changes in metagenomic linkage group (MLGs) (95). Therefore, the drug may affect the microbiome by affecting the expression of genes in gut microbiome. As mentioned above, metoprolol therapy seems to change the intestinal microbiome, indicating that metoprolol may directly or indirectly affect gut microbiota composition.

### Avenues Toward Gut Microbiome-Based Precision Medicine

The gut microbiota, like any ecosystem, is profoundly complex. The composition and number of specific gut microbiota vary among individuals and can be changed rapidly, such as different dietary structures and environmental changes (high altitude hypoxia), as well as the combined use of drugs (antibiotics and antihypertensive drugs) (54, 65, 96). These factors may change the initial state of gut microbes. Since the gut microbiota is highly individualized (97), individuals' gut microbiomes may have different effects on absorption, distribution, and metabolism of certain drugs. Consequently, the gut microbiota presents a significant potential for personalized treatment (98, 99), including antihypertensive response biomarker, microbial-targeted therapies, probiotics therapy, etc.

### Gut Microbiota Is a Potential New Territory for Individualization and Precision Medicine

The importance of gut microbiome in drug therapy of hypertension indicates a precision medical approach driven by the microbiome, explicitly targeting the microbiome to obtain clinical results and improve clinical efficacy (24). Firstly, the gut microbiome is considered a response biomarker. Predicting the efficacy and toxicity of antihypertensive drugs in the body by measuring composition and quantity of gut microbiome and relative abundance of its metabolites. Fecal microbial characteristics may also be used as a non-invasive diagnostic method or provide prognostic evaluation. Secondly, designing small molecules to inhibit the activity of microbial metabolism *in vivo* related to biotransformation of drugs into toxic metabolites (99). For example, research is currently underway to inhibit TMAO precursor, trimethylamine, a small molecule produced by gut microbiome regulating blood pressure (100, 101). Finally, since new technologies now identify microbial communities, strains, or metabolites that can affect drug efficacy, the microbial composition can be modified by introducing natural or engineered products such as probiotics (99). Adjusting the composition and ratio of microbiome can improve the bioavailability of drugs and reduce adverse reactions.

In the era of precision medicine, gut microbiota has made great progress in the role of allogeneic hematopoietic cell transplantation (102). The diversity and stability of the gut microbiota are important indicators for identifying patients at risk for graft vs. host disease (GVHD) and adverse outcomes (103), and may become a biomarker of GVHD, predicting the recurrence, infection and transplant-related mortality of GVHD (104). Therefore, gut microbiota may become a new strategy to improve the prognosis of allogeneic hematopoietic cell transplantation in the future, such as the study of fecal microbiota transplantation, prebiotics administration, the use of different antibiotics, intestinal decontamination and the effect of E.limosum in relapse effect (25, 105).

### The Therapeutic Effect of Probiotics

The effects of probiotics on human health have sparked widespread interest. Probiotics have been widely used in food and even pharmaceutical fields for decades (106). Probiotics are defined as “live microorganisms, if used properly, can bring health benefits to the host” (107, 108). As a result, it is unsurprising that probiotics are a critical tool for influencing human health and disease via gut microbiome balance (109).

Researchers have found that probiotics are beneficial in treating hypertension, dyslipidemia, lactose intolerance, obesity, heart failure and myocardial infarction (110–112). For instance, probiotic strains and probiotic fermented foods have great benefits for regulating blood pressure based on *in vivo* studies (113–115). Numerous studies have shown probiotics can reduce systolic or diastolic blood pressure (SBP/DBP) (Table 2). In a randomized, double-blind, placebo-controlled study, consumption of Lactobacillus helveticus fermented milk product by 46 borderline hypertensive men reduced SBP 5.2 mmHg and DBP 2.0 mmHg (123). A mean reduction of SBP 13 mmHg and DBP 5 mmHg has been recorded in 36 smokers (aged 35–45 years) given fermented food with Lactobacillus plantarum 299v for 6 weeks (117). In addition, probiotics strains such as *Lactobacillus casei, Lactococcus spp, Lactobacillus plantarum* and *Streptococcus thermophilus* can also reduce SBP (124, 125). Probiotics can regulate blood pressure through several different mechanisms. For example, lactic acid bacteria can metabolize milk protein and help release short bioactive peptides with ACE-inhibitory activity, thereby regulating hypertension (126). Lactobacillus johnsonii La1 might lower blood pressure by changing autonomic neurotransmission of the central histaminergic nerve and suprachiasmatic nucleus in rats (127).

**Table 2:** The effect of probiotics in the regulation of blood pressure.

**Probiotic strains**	**Subjects (No. of intervention/ control)**	**Age range (year)**	**Dose (CFU/day)**	**Time, week**	**Form**	**Study design**	**Intervention baseline (changes from baseline)**	**Control baseline (changes from baseline)**	**Refs**
Lactobacillus. helveticus; Saccharomyces. cerevisiae	30 hypertensive patients (17/13)	40–86	7.0 ×10^11^; 2.5 ×10^9^	8	Fermented milk	Randomized, placebo-controlled	SBP: 158.5 (↓ 14.1) DBP: 88.7 (↓ 6.6)	SBP: 150.9 (↓ 4.4) DBP: 87.6 (↓ 2.2)	(116)
Lactobacillus. plantarum 299v	36 healthy volunteers (18/18)	35–45	5 ×10^7^	6	Rose-hip drink	Randomized, double-blind, placebo-controlled	SBP: 134 (↓ 13) DBP: 89 (↓ 5)	SBP: 128 (↓ 2) DBP: 89 (↓ 4)	(117)
Lactobacillus. plantarum TENSIA	40 hypertensive patients (25/15)	30–69	1.5 ×10^11^	3	Probiotic cheese	Randomized, blinded-controlled	SBP: 134 (↓ 12.2) DBP: 82.4 (↓ 8.0)	SBP: 131.4 (↓ 11.4) DBP: 82.1 (↓ 3.5)	(118)
Bifidobacterium.lactis; Lactobacillus. acidophilus	38 healthy men (20/18)	18–54	1 ×10^9^	4	Yogurt	Randomized, double blind, placebo-controlled	SBP: 104.6 (↓ 2.5) DBP: 70.2 (↓ 0.9)	SBP: 103.8 (↓ 0.8) DBP: 69 (+ 1.3)	(119)
Enterococcus. faecium; Streptococcus. thermophilus	30 healthy volunteers (16/14)	18–55	4.7 ×10^11^	8	Yogurt	Randomized, double-blind, placebo- controlled	SBP: 131.9 (↓ 8) DBP: 83 (↓ 4)	SBP: 116.5 (↓ 2.2) DBP: 76.4 (↓ 1.5)	(120)
Streptococcus. thermophilus; Lactobacillus. Acidophilus; Bifidobacteria infantis	101 healthy volunteers (53/48)	20–60	4.8 ×10^12^	8	Yogurt	Randomized, double-blind, placebo-controlled	SBP:110.2 (↓ 1.07) DBP:70.7 (↓ 0.32)	SBP: 110.9 (↑ 0.91) DBP: 71.6 (↓ 0.43)	(121)
Lactobacillus sporogenes	54 diabetic patients (27/27)	35–70	1 ×10^8^	8	Bread	Randomized, double-blind, placebo-controlled	SBP: 143.9 (↓ 6.4) DBP: 87.1 (↓ 3.8)	SBP: 145.0 (↓ 5.7) DBP: 92.3 (↓ 5.2)	(122)

*SBP, systolic blood pressure; DBP, diastolic blood pressure*.

As mentioned above, the antihypertensive function of probiotics will make it possible to become antihypertensive drugs (114). To our knowledge, certain synthetic antihypertensive drugs may cause adverse reactions, such as coughing, dizziness, angioedema, headache and indigestion (128, 129). Compared with conventional drugs, probiotics reduce the adverse effects and provide new treatment options for hypertension. Considering that hypertension pathophysiological mechanisms could vary between patients, we can choose specific probiotics to gain benefits in a specific hypertensive patient (130). In addition, probiotics might also be combined with antihypertensive drugs to assist drug treatment and better exert the effects of drugs. Probiotics interact with original microbial community *in vivo* to maintain intestinal flora homeostasis and affect the related enzyme activity, thereby change pharmacological activity and bioavailability of drugs (131). Precise control of intestinal flora may be used as a treatment option, either alone or combined with other therapeutic targets. This treatment strategy is very novel and attractive. It targets microorganisms and their metabolism rather than the host. Compared with drugs that target host metabolism, the intestinal flora is much less likely to have side effects (132).

### Fecal Microbiota Transplantation

Fecal microbiota transplantation (FMT) is a therapeutic intervention that aims to transplant the gut microbiota obtained from the feces of a healthy donor into the patient's gastrointestinal tract, gaining therapeutic enefit (133). Most often, this therapy is used to treat gastrointestinal diseases caused by the activity of pathogenic or conditional pathogenic microorganisms, including Clostridium difficileinfection, and inflammatory bowel disease (134). Mounting evidence indicates that FMT also treats metabolic syndrome, diabetes, and hypertension (94, 135). Suez et al. compared the effects of probiotics and FMT methods in rebuilding the gut microbiome of mice and humans, and found that FMT aided in quickly and thoroughly restore the human gut microbiome and host intestinal transcriptome compared with probiotic supplementation (136). Therefore, the use of FMT to reshape the gut microbiome is an attractive strategy to restore hostmicrobiota crosstalk (137). For example, the FMT from SHRs to normal rats increased the SBP of normal rats. Conversely, FMT from normal rats to SHR reduced the SBP of SHRs (138, 139). In addition, FMT from normotensive rats to SHRs not only improved blood pressure, but also improved vasodilation function (138). FMT is now on the edge of a new era. Despite the therapeutic promise of FMT, its application to precision medicine requires overcoming considerable hurdles. Therefore, we need to more accurately characterize the characteristics of patients and donors to determine who, when and how doctors should provide FMT.

## Conclusion

Research into the gut microbiome has opened up a whole new approach for precision treatment of hypertension. We have described the effective interactions between commonly used antihypertensive drugs and the gut microbiome. The gut microbiome affects the drug metabolism by changing their chemical structures. Simultaneously, the gut microbiome composition and function may also be changed in the process of drug metabolism. When combining clinical medications, clinicians need to combine this factor to use medications more rationally. Given the individual differences of the gut microbiome and the complex drug-microbiota interactions, we need to further understand the underlying causality and mechanisms. In the field of precision medicine, the specific mechanisms and clinical trials of the interaction between microorganisms and drugs will be deeply studied, and the results will affect clinical practice in the foreseeable future.

## Author Contributions

J-QL and HR: conceptualization and supervision. H-QC: writing—original draft preparation. J-QL, J-YG, M-ZL, and KX: review and editing. All authors have read and agreed to the published version of the manuscript.

## Funding

This work was supported by Wu Jieping Medical Foundation (320.6750.2020-04-14), National Natural Science Foundation of China (No. 81703623), and the Scientific Foundation of Hunan (No. 2018JJ3719).

## Conflict of Interest

The authors declare that the research was conducted in the absence of any commercial or financial relationships that could be construed as a potential conflict of interest.

## Publisher's Note

All claims expressed in this article are solely those of the authors and do not necessarily represent those of their affiliated organizations, or those of the publisher, the editors and the reviewers. Any product that may be evaluated in this article, or claim that may be made by its manufacturer, is not guaranteed or endorsed by the publisher.
